# Morphological Transformation of Silver Nanoparticles from Commercial Products: Modeling from Product Incorporation, Weathering through Use Scenarios, and Leaching into Wastewater

**DOI:** 10.3390/nano9091258

**Published:** 2019-09-05

**Authors:** Selvan Mohan, Juliska Princz, Banu Ormeci, Maria C. DeRosa

**Affiliations:** 1 Department of Chemistry, Carleton University, 1125 Colonel by Drive, Ottawa, ON K1S 5B6, Canada; 2 Environment and Climate Change Canada, 335 River Road South, Ottawa, ON K1V 1C7, Canada; 3 Department of Civil and Environmental Engineering, Carleton University, 1125 Colonel by Drive, Ottawa, ON K1S 5B6, Canada

**Keywords:** nanoparticle, Silver (Ag), life cycle, TEM, EDS, ICP-MS, XPS

## Abstract

There is increasing interest in the environmental fate and effects of engineered nanomaterials due to their ubiquitous use in consumer products. In particular, given the mounting evidence that dramatic transformations can occur to a nanomaterial throughout its product lifecycle, the appropriateness of using pristine nanomaterials in environmental testing is being questioned. Using a combination of transmission electron microscopy (TEM), energy dispersive X-ray spectroscopy (EDS), X-ray photoelectron spectroscopy (XPS), and inductively coupled plasma-mass spectrometry (ICP-MS), this work examines the morphological and compositional effects of conditions mimicking a typical lifecycle of a nano-enabled product, from the production of the silver nanoparticle (AgNP)-laden textiles, through its use, laundering, and then finally, its leaching and incubation in the wastewater collection system. These simulated weathering conditions showed evidence for the transformation of AgNPs into AgCl and Ag_2_S. Incubation in raw wastewater had the most dramatic effect on the AgNPs in terms of transformation, no matter what initial weathering was applied to the NPs prior to incubation. However, despite extensive transformation noted, AgNPs were still present within all the samples after the use scenarios.

## 1. Introduction

The unique properties of nanoparticles (NPs) make them attractive additives in a variety of commercial products. With the heightened interest in this area of research has come a plethora of novel nanomaterials of varying composition and morphology, many with attractive possible applications [1,2]. Of the over 1600 products claiming to contain nanomaterials, 383 of them explicitly declare the incorporation of silver [3]. Although AgNPs are applied to many different commercial products worldwide, the majority of them are used in textiles for antimicrobial purposes [4,5,6,7]. The ubiquity of AgNP-laden products in the marketplace sparks the question of the eventual environmental fate and effects of these materials.

Studies on the environmental impact of nanoparticles are typically carried out using the material in pristine form and vary widely in size (10–50 nm) and coatings depending on the availability and ease of synthesis of the nanoparticles [8,9,10,11,12,13,14]. Numerous studies have established the potential ecotoxicological effects of AgNPs. They inhibit the photosynthesis of algae, increase the mortality rate of aquatic zebrafish embryos, and disrupt microbial colonies in soil [14,15,16,17,18,19,20,21]. While an understanding of the potential impact of pristine nanoparticles on the environment is essential, particularly in simulating accidental spill scenarios, these results might not be universally applicable to all exposure situations, given the transformations that NPs could undergo throughout their life cycle [22]. Transformations can include the agglomeration or aggregation of the nanoparticle or a chemical/surface transformation triggered by exposure to other agents within the product or exposed to the product. Although often used interchangeably, aggregation is defined as the loose assembly of particles and agglomeration is the more permanent assembly of the particles into tight clusters causing partial fusion [23]. Several studies have already established that transformations to AgCl or Ag_2_S are possible for AgNPs [24]. This transformation has been previously described as oxidative dissolution by chlorine and sulfidation, respectively [25,26]. However, the presence of chloride, nitrate, selenide, and sulfide have also been known to lead to AgNPs transformations [27,28,29]. Indeed, analyzing transformed NPs and subsequent byproducts released from the use of products containing NPs will inform environmental fate and toxicological studies, as well as risk assessment initiatives [30].

With regards to Ag, studies have shown that AgNPs are released from AgNP-laden fabric using only water washes [31,32]. Treatments with sweat and detergent have also been shown to trigger the release of NPs [33,34,35]. The composition, pH, and ionic strength of these solutions will contribute to the variability in the form of Ag extracted [24]. As the fabrics are exposed to these conditions (e.g., washing, sweat.), the potential is high that the leachates from the consumer product, containing transformed AgNPs, will eventually end up in the wastewater treatment process [36]. However, even before reaching the wastewater treatment facility, incubation in the wastewater collection system could further transform the nanomaterials. Pristine nanoparticles are known to disrupt microbial colonies, biofilms, and alter the chemical properties of the wastewater, hindering the effectiveness of the wastewater treatment [37,38,39]. Further transformation of these AgNPs can be expected through the wastewater treatment, as well as during environmental weathering [40,41,42]. Finally, sludge from wastewater is treated for use in agricultural applications [43]. The transformation of AgNPs to Ag_2_S when added directly to the sludge, had been reported [44]. Ag_2_S nanoparticles of 5 to 10 nm have also been independently detected in sewage sludge [45].

**Figure 1:**
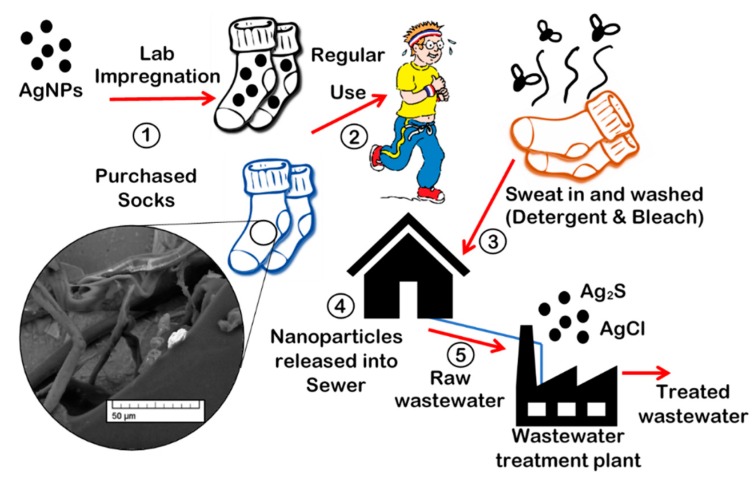
Overview of the path of potential transformations for silver nanoparticles (AgNPs) studied in this paper.

A clearer understanding of the physical and chemical changes that take place throughout the lifecycle of a nano-enabled product would help inform toxicological studies and improve our risk assessment for nanomaterials. In this paper, the morphology and composition of AgNPs, when embedded into fabric, are compared following a variety of treatments meant to mimic several stages in the lifecycle of the nanomaterial, from initial product incorporation to use and washing, to right before the wastewater treatment process (Figure 1). 

## 2. Materials and Methods 

**Figure 2:**
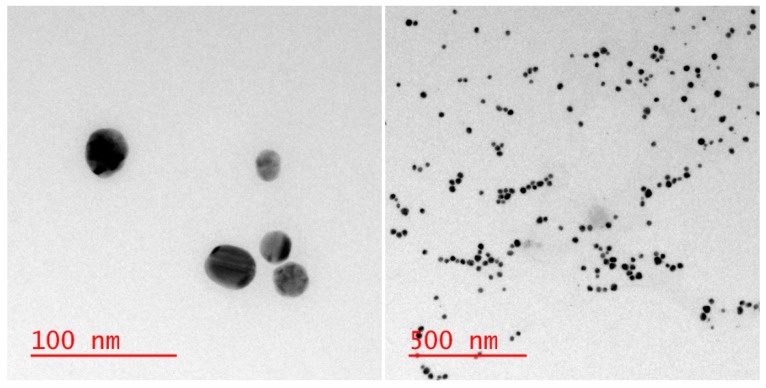
TEM images of 25 nm pristine AgNPs from Nanocomposix in double deionized water (ddH_2_O). Left: Close up view showing particles of mostly spherical shape. Right: A full view of the nanoparticle sample, indicating a reasonably monodisperse sample with little to no aggregation.

Pristine AgNPs, 25 ± 3 nm Econix Silver Nanospheres (5 mg/mL concentration, −23 mV zeta potential) were purchased from Nanocomposix, San Diego, CA, USA (Figure 2). Socks with AgNPs (Sports Socks) were purchased from Nanosilver, Olomouc, Czech Republic (commercial socks). White Hanes sport cut socks (Winston-Salem, NC, USA), Clorox^®^ bleach (Oakland, CA, USA) and Tide® Original laundry detergent (Procter & Gamble, Cincinnati, OH, USA) were used as received. Raw wastewater (filtered to remove large solid matter) was obtained from the influent of the Robert O. Pickard Environmental Centre wastewater plant (Ottawa, ON, Canada). Transmission electron microscopy (TEM) grids were purchased from Electron Microscopy Sciences, Hatfield, PA, USA. All acids used were ultrapure reagent grade unless specified. Only double deionized water (ddH_2_O) was used in this experiment. All other chemicals were purchased from Sigma Aldrich (MilliporeSigma Canada Co., Oakville, ON, Canada) and used as received. 

### 2.1. Preparing Socks for In-Situ Impregnation (Kier boiling)

Kier boiling of the commercial socks was employed to remove the wax finishing and non-cellulosic materials on the cotton fibers [46]. In brief, 14.7 g of Na_2_CO_3_ and 33.3 grams of NaOH were added to 1 L of ddH_2_O in a 2 L round bottom flask. A pair of white Hanes socks was added to the solution and refluxed for 10 h. The solution turned yellow when boiling and eventually turned brown, indicating the removal of waxy and non-cellulosic material [46]. The socks were then rinsed five times in a 20% bleach solution and left to air dry.

### 2.2. In-Situ Impregnation of Ag Nanoparticles into Socks (Laboratory-Prepared Socks)

A kier boiled sock was immersed in 1 mM AgNO_3_ solution in a 4 L beaker at 1:20 (*w*/*v*) and then autoclaved for 15 min at 121 °C and 15 psi. Thereafter, the solution was left to cool to room temperature [47]. The socks were then rinsed with ddH_2_O (3 × 1000 mL) to remove any free NPs and then air-dried. The socks turned from white to brown, indicating the deposition of AgNPs on the socks [47].

### 2.3. Day-to-day Wear with Sweat, Bleach, and Detergent 

Each treatment comprised a single sock in a 1 L beaker with 750 ml of one of three solutions mimicking typical product use. For the sweat solution, a 1.08% NaCl, 0.12% lactic acid, and 0.13% urea solution was prepared (pH 3) [34]. To mimic typical bleaching or laundering conditions, 10 mL of bleach in 750 mL of water (pH 11.3), and 6 mL of detergent in 750 mL of water (pH 8.6) were used, respectively [31]. The solutions were then agitated on an Innova 40 Incubator Shaker (Eppendorf Inc., Enfield, CT, USA) at 100 oscillations per minute at 37 °C for 24 h, to simulate multiple uses and wash cycles. 

### 2.4. Wastewater Exposure 

Raw wastewater was characterized (see Supporting Information, Table S1) and used the very same day unless indicated. Five microliters of the pristine NPs solution (5 mg/mL) was added to 2 mL of raw wastewater. The leachate from the bleach, detergent, and sweat solutions was each mixed separately with wastewater at a 1:1 ratio to make up a 2 mL solution. The mixtures were then vortexed on a Vortex-Genie 2, (Scientific Industries Inc., Bohemia, NY, USA) for 10 minutes, and then left for 24 h at 21 °C before the TEM grids were prepared.

A 15 mg/L aqueous solution of 1,6-hexanedithiol and a 5 mg/L aqueous solution of ammonium hydroxide were also used as model sulfur-rich and nitrogen-rich solutions, respectively. Pristine NPs and leachate from the bleach, detergent, and sweat solutions were directly added to the sulfur or nitrogen solutions, vortexed for 10 min and left for 24 h at 21 °C.

### 2.5. Sample Preparation for ICP-MS Analysis

#### 2.5.1. Incineration

A small pre-weighed piece of the sock (~100 mg) was placed in a 10 ml Pyrex beaker and placed into a TEMCO 1520 benchtop muffle furnace. The furnace was heated to 600 °C and left for 3 h or until the sample turned to white ash. The sample was let to cool to room temperature before removing from the furnace [34]. The ash was then dissolved in 100 µL HNO_3_ and diluted 10X with ddH_2_O.

#### 2.5.2. Aqua-Regia

A small pre-weighed piece of the sock (~100 mg) was soaked in 1:3 ratio of HNO_3_ and HCl for 1 h and then heated to 90 °C for 2 h [48]. The sample was diluted 10X with ddH_2_O before analysis.

#### 2.5.3. HNO_3_/H_2_O_2_

A small pre-weighed piece of the sock (~100 mg) was submerged in 1:1 ratio of HNO_3_ and H_2_O. The solution was heated to 100 °C, and more HNO_3_ was added slowly until most of the sock dissolved. The solution was allowed to cool, and 30% H_2_O_2_ was added until the bubbling stopped [31]. The sample was diluted 10× with ddH_2_O before analysis.

### 2.6. Analysis

ICP-MS was performed on an Agilent 8800QQQ (Agilent, Santa Clara, CA, USA) or an Agilent 7700x ICP-MS (Agilent, Santa Clara, CA, USA) (the latter used only to determine the best extraction method). The ICP-MS samples for the socks were prepared by dissolving the ash after incineration in 1 mL HNO_3_ and diluting that in 9 mL ddH_2_O. The solutions from the extractions from Section 2.5, leachates, and wastewater incubations were used as-is. Appropriate dilutions were made to ensure the Ag concentration was within the calibration curve, and the results were normalized to compare the extraction methods.

The TEM images were taken using FEI Tecnai G2 TEM (Thermo Fisher Scientific, Waltham, MA, USA) at 200 kV. Samples were first prepared the same way as for the ICP-MS. The solutions were then vortexed for two minutes, and thereafter, 6 µL of sample was placed on the grid and left for a minute. Residual liquid was removed using a pipette, and another 6 µL of sample was added. The residual sample was removed for the second time, and the grid was left to air dry for 2 h. The CF300-Cu grid was used.

The EDS of the sample was taken through an attachment to the TEM, Aztec EDS from Oxford X-ray detection systems (Oxford Instruments, Tubney Woods, Abingdon, UK). The beam was made to top focus directly on the nanoparticle.

The XPS were taken using Kratos analytical model Axis Ultra DLD using AlK_α_ radiation, charge neutralizer, and a delay-line detector (DLD) (Kratos Analytical Ltd, Wharfside, Manchester, UK). The samples used for XPS were all in the liquid form wherein 10 µL of the sample was applied onto a glass coverslip. The sample was left to air dry for 2 h. Once dried, another 10 µL of sample was applied to the exact same spot and again left to air dry for 2 h. This step was repeated 10 times or more until a visible spot was observed on the glass coverslip.

## 3. Results and Discussion

**Figure 3:**
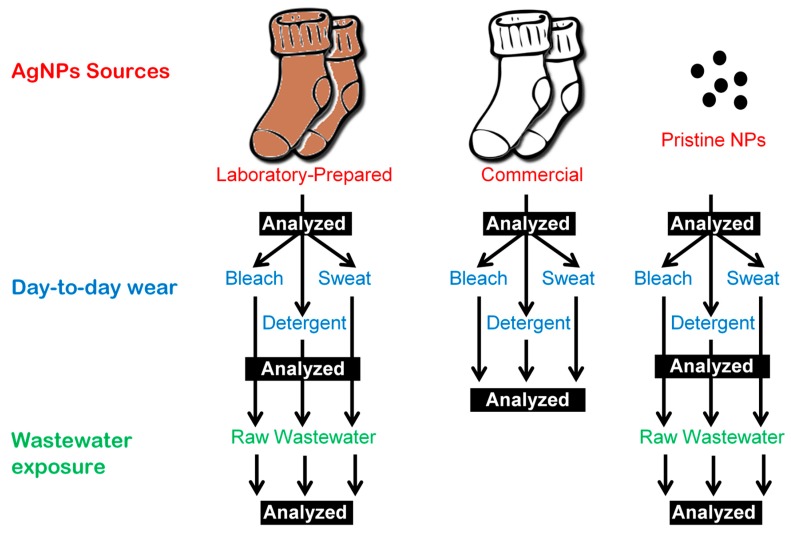
Summary of the lifecycle path and analysis of different AgNP sources studied in this paper. All analyses consist of TEM, EDS, and ICP-MS. The commercial socks were only analyzed through ICP-MS.

The diagram in Figure 3 shows the experimental design and path taken in analyzing the transformations of AgNP from three different sources through different use scenarios. The sources of AgNPs studied were the commercial socks, laboratory-prepared AgNP socks (in-situ NP synthesis used in industry [49,50]) and pristine AgNPs.

**Figure 4:**
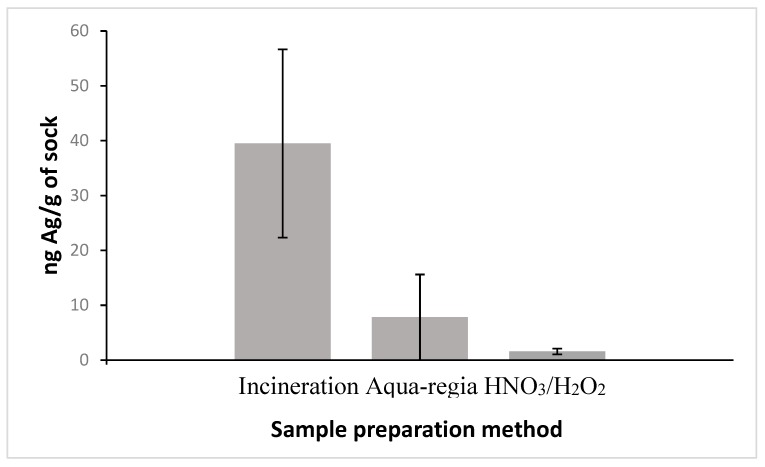
Mean (± standard deviation) total Ag, derived from five samples, as measured through ICP-MS, based on different Ag extraction procedures on commercial socks before day-to-day use and wastewater exposure.

However, to analyze the silver content in these socks, either prior to and subsequent to use and wastewater exposure, a suitable method of extraction was required. Three methods (incineration, aqua-regia digestion, and nitric acid/hydrogen peroxide digestion) were selected, and the effectiveness of each technique was evaluated by ICP-MS results. The background Ag concentration of the instrument was measured to be 5 ng/L for the ICP-MS. Figure 4 shows the effectiveness of each preparation method in extracting Ag from the same commercial sock before day-to-day wear and wastewater exposure. Incinerating the socks yielded the most Ag extracted, confirmed by ANOVA statistical test (F > F crit, P = 0.000294). As a result, incineration was selected as the preferred method of analysis and subsequently used to prepare the remainder of sock samples for analysis. 

**Figure 5:**
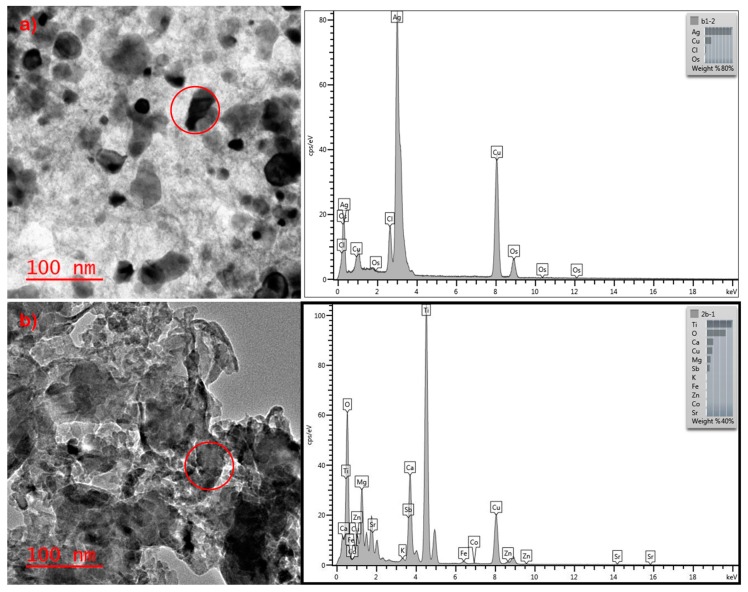
TEM and EDS image of (**a**) AgNPs found in laboratory-prepared socks (**b**) Only TiO_2_ and ZnO NPs found in commercial socks.

Transmission electron microscopy with Energy Dispersive X-ray Spectroscopy (TEM-EDS) was used throughout this study to confirm the presence of AgNPs and to note morphological and compositional changes. However, despite being able to quantify Ag in these textiles by ICP-MS, the AgNPs could not be found in the commercial socks through TEM and EDS analysis, as shown in Figure 5. The NPs found in the TEM images of commercial socks were determined to be a majority of TiO_2_ NPs. TiO_2_ NPs can also be added as a white pigment within fabrics to mask the brown color of AgNP [51]. The overwhelming presence of TiO_2_ may have masked the ability to detect Ag through TEM-EDS. In contrast, the AgNPs could readily be observed in the laboratory-prepared socks with a size distribution of between 5 to 50 nm via TEM-EDS. The laboratory-prepared socks were brown as no TiO_2_ oxide was used to mask the color.

The Ag-containing socks (commercial and laboratory-prepared socks) were then analyzed for Ag content through ICP-MS, and the results are shown in Table 1. The Ag content of the laboratory-prepared sock was about 5000× higher than that of the commercial socks. This low Ag content in the commercial socks was below the detection limit for TEM-EDS. However, the concentration of Ag in the commercial sock is consistent with what has been reported in the literature; 2–1360 µg-Ag/g-socks was detected when analyzing five different Ag-containing commercial socks [31]. Further analysis of the commercial socks was only done by ICP-MS as the detection of Ag in the commercial socks was not possible via TEM-EDS. 

Three systems were evaluated through simulated day-to-day use conditions (sweating, washing, and bleaching): the commercial and laboratory-prepared socks, as well as free, pristine AgNPs (Figure 3). Human sweat varies in its pH and composition from different individuals. Kulthong et al. (2010) evaluated the effect of artificial sweat of different pH ranges on both laboratory-prepared and commercial fabrics and determined that a pH of 6.5 released the most amount of AgNPs [34]. A similar synthetic sweat composition was, therefore, used in this study [34]. Commercial detergent and bleach were also used in the present study, and concentrations followed that prescribed for use by the commercial products to mimic realistic washing and bleaching conditions. The pH of the bleach, detergent, and sweat solution were 11.3, 8.6, and 2.8, respectively. Another study had shown pH to not affect the chemical properties of AgNPs [52]. Exposing AgNPs to solutions of pH 2 to 10 did not change the zeta potential of the nanoparticles [52].

**Figure 6:**
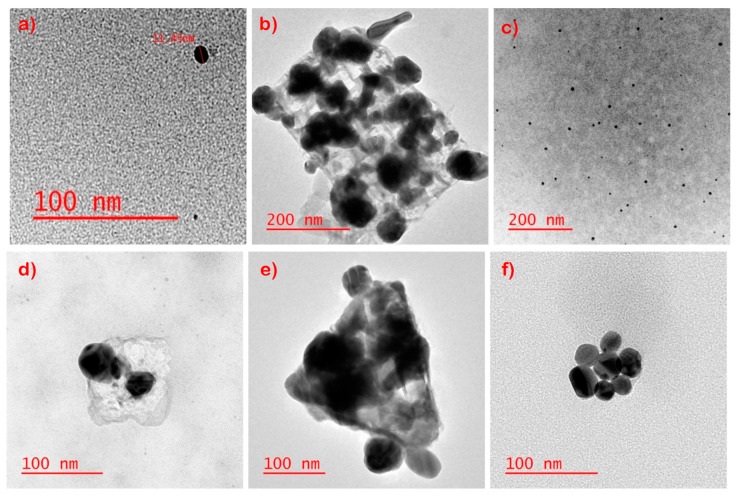
Representative TEM images of laboratory-prepared socks weathered with of (**a**) sweat, (**b**) bleach, (**c**) detergent and TEM images of pristine NPs weathered with (**d**) sweat, (**e**) bleach, and (**f**) detergent. See Supplementary Materials Figures S1 and S2 for EDS data.

**Figure 7:**
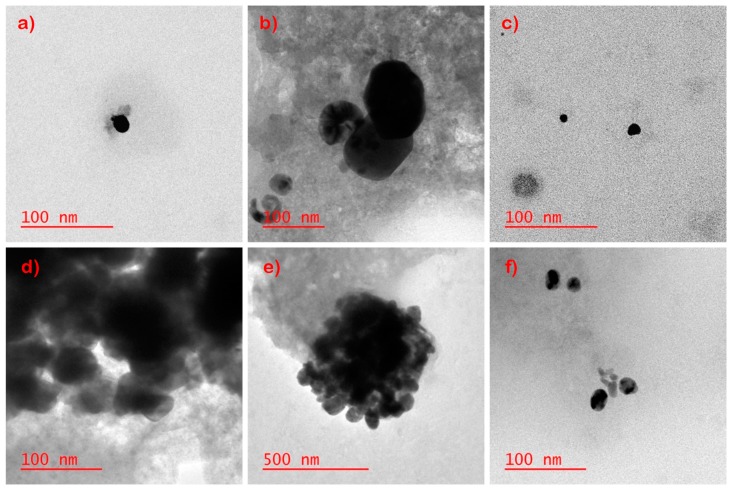
Representative TEM images of (**a**) sweat, (**b**) bleach, (**c**) detergent solution from laboratory-prepared socks and TEM images of (**d**) sweat, (**e**) bleach, (**f**) detergent solution from pristine NPs after 24-hour incubation with wastewater. See Supplementary Materials Figures S3 and S4 for EDS data.

The commercial and laboratory-prepared socks were incubated with bleach, detergent, and sweat solutions for 24 h to mimic day-to-day wear. These solutions were then analyzed using ICP-MS, of which results are shown in Table 2. Both the detergent and sweat solution had extracted Ag from the commercial socks, but there was no significant Ag in the bleach solution. The absence of Ag in the bleach solution could be due to the higher pH of the solution, limiting the extraction of Ag from the cotton fibers, or the extracted NPs could have agglomerated and settled leading to an inhomogeneous sample for ICP-MS. Such large agglomerated Ag particles caused by the bleach solution were observed in TEM images of samples (Figure 6 and Figure 7). The observed agglomeration of Ag can be due to the oxidative dissolution of AgNPs, followed by the precipitation of AgCl [25].

Since the initial concentrations of Ag were low, TEM images were not taken for any of the solutions from the commercial socks. The laboratory-prepared socks had much more silver extracted into solution. However, the ratio of Ag released compared to the total Ag content in the socks was similar between the commercial and laboratory-prepared socks, indicative that the binding strength between the AgNPs and the cotton fibers in the lab-prepared socks resembled the commercial socks. Having similar nanoparticle release ratio makes the laboratory prepared socks a suitable model to yield an accurate representation of the commercial socks for day-to-day use and wastewater exposure.

Selected TEM images of the extracts from the laboratory-prepared socks sample exposed to bleach, detergent, and sweat conditions are shown in Figure 6a–c. Apart from the bleach solution, the AgNPs are seen to remain well-dispersed in the extracted solutions. The bleach solutions showed aggregated assemblies, appearing to be nucleated on salt crystals. EDS indicated the association of S and Cl with AgNPs in the sweat and detergent extracts, while only Cl could be co-detected with Ag in the bleach extracts.

For comparison, pristine NPs were put through the same conditions as the laboratory-prepared socks (Figure 6d–f). In the sweat solution, the AgNP were mostly well dispersed, and some association on NaCl salt crystals was observed. The AgNPs extracted from the bleach solution appeared to once again to be aggregated, and Cl could be co-detected by EDS. Finally, in detergent, small aggregates could be observed with only S co-detected. The pristine AgNPs show a similar transformation as the AgNPs that leach out of the laboratory-prepared socks (Figure 6a–c).

AgNPs extracted from a textile by sweat, bleach, or detergent could find their way into the wastewater collection system by bathing of the user or washing of the textile. It is estimated that wastewater travels within the collection system for a United States’ national average of 3.3 h up to as long as 15 h in larger cities before reaching the wastewater treatment facility [53]. During this time, the NPs will be exposed to organic matter, nitrogen, phosphorus, sulfur, metals, and a wide range of chemical compounds and microorganisms. The chemical and microbial reactions, dissolved oxygen concentration, oxidation-reduction potential, ionic strength, and pH of wastewater will change as a function of residence time in the sewer, which can physically and chemically transform the AgNPs. The effect of incubation in raw wastewater was compared for the AgNPs that had been extracted through the various use-scenarios, as well as pristine NPs, to observe the effects on morphology or composition.

The solutions obtained from bleach, detergent, and sweat extraction of laboratory-prepared socks were added 1:1 to wastewater to observe the effects on the AgNPs. (Figure 7). With the bleach solution, the AgNPs agglomerated as their average diameter increased from 5 to 50 nm to >100 nm. In contrast, the particle sizes detected from the detergent and sweat solutions post-wastewater incubation did not show an increase of particle size. However, halos with high S and Cl content could be observed around the dispersed particles suggesting a respective association with Ag and potentially dissolution and re-precipitation.

The solutions obtained from bleach, detergent, and sweat of pristine NPs were also examined after a 24 h incubation in wastewater (Figure 7). Larger aggregates and agglomerates with low levels of Cl content could once again be observed in the bleach sample. NPs in the sweat solution appeared to be similar to those in the bleach solution. The detergent samples appeared more dispersed, and significant association with Cl and S was observed.

To have a better understanding of the effect of wastewater composition on AgNP morphology and composition, pristine AgNPs that had not undergone any pretreatment were introduced to the wastewater, as well as sulfur-rich and nitrogen-rich solutions, and 1:1 wastewater/sulfur solution and wastewater/nitrogen solutions (Supplementary Materials Figure S5). Pristine AgNPs incubated in wastewater became aggregated, and sulfur-rich halos were observed around the particles. The introduction of the AgNPs to the model sulfur-rich solutions showed similar morphology effects to that observed with wastewater, while exposure to our nitrogen-rich solution did not lead to aggregation or show formations of halos. The detection of nitrogen could be because of ammonia forming a stable ligand with Ag, $\mathrm{Ag}{\left({\mathrm{NH}}_{3}\right)}_{2}^{\;+}$ which is stabilized by the OH^−^ ions in solution [54]. Similar results were observed by adding pristine NPs to mixtures of wastewater + sulfur-rich solution and wastewater + nitrogen-rich solution. The effects of different wastewater pH (4.0, 6.0, and 8.0) were also tested (Supplementary Materials Figure S6). The change in pH did not affect the form or aggregation state of AgNPs. Pristine nanoparticles have been shown to start aggregating when the pH drops below pH 3.0 [52].

**Figure 8:**
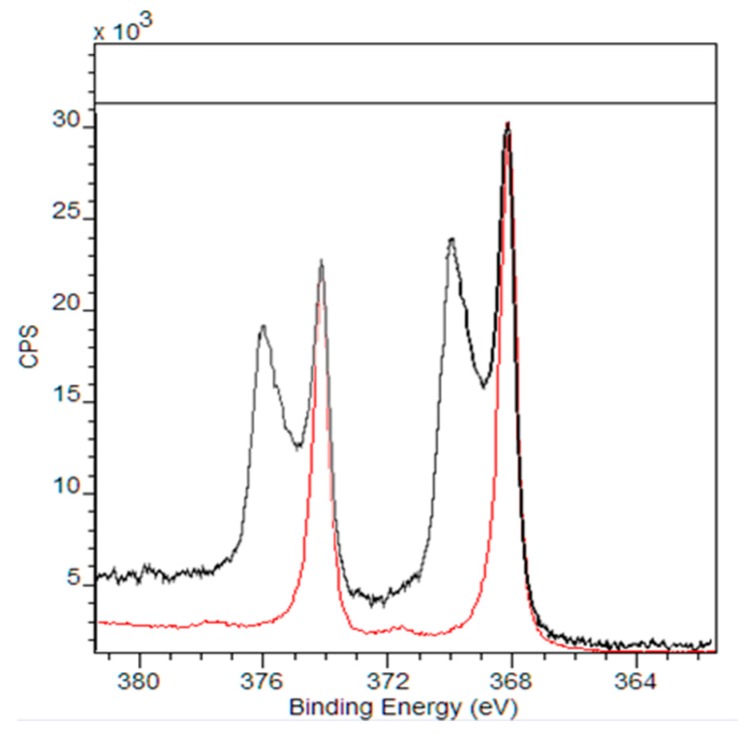
XPS spectra showing the binding energy of Ag of pristine AgNPs (red) and those incubated in wastewater (black) obtained through high-resolution XPS.

To further analyze these chemical changes, samples of pristine AgNPs incubated in wastewater were analyzed using XPS. In Figure 8, a shift in binding energy was observed for Ag when the nanoparticles were incubated with wastewater. The shift to higher binding energy is caused by the oxidation of the Ag atom [55]. The shift of the binding energy further supports the association of S and Cl with AgNPs observed in EDS are due to the AgNPs going through a chemical transformation [56].

Table 3 below shows the summary based on all the TEM images and EDS data from the samples. The nanoparticles were observed to aggregate, agglomerate, and associate with chlorine and sulfur when exposed to wastewater, regardless of origin and previous weathering. Images shown in this paper were selected as being the best representation of the AgNPs on the 100 nm scale. Some agglomerations were observed to reach the micro scale, which supports the recent reports where pristine AgNP exposed to simulated detergent and bleach wash cycles form large agglomerates [33,57]. The elemental association with Cl and S observed with EDS is likely because of the formation of AgCl and Ag_2_S. A similar interpretation of the EDS data for AgNPs in simulated wash cycles had been reported in a recent study [35].

However, not all the nanoparticles imaged and analyzed appeared to have oxidized to AgCl and Ag_2_S. According to a previous study on introducing commercial AgNP fabric to the bleach and detergent, only 50% of the AgNPs were converted to AgCl. [58] Since X-ray Absorption Near Edge Spectroscopy (XANES) was used for elemental analysis in that study, only the surface of the nanoparticles were analyzed for the presence of AgCl. The percent AgCl would potentially be lower if the entire core of the nanoparticle was analyzed [59]. 

Despite the qualitative nature of this study, some important conclusions can be drawn about the transformations of AgNPs during these simulated day-to-day use and wastewater exposure processes. While AgNPs do not retain their pristine form from product incorporation through use scenarios, to travel in the wastewater collection stream, they are also not fully dissolved throughout these steps. Given that others have detected AgNPs in the wastewater influent and effluent as well as in sewage sludge, our use of these simulated scenarios represents the real-world scenarios of the nanoparticle life-cycle [45,60]. Furthermore, despite evidence for transformations throughout the use and exposure steps, the most dramatic changes to the nanoparticle morphology and composition appear to have occurred in our final step mimicking the time spent in the wastewater collection stream. As a result, using a 24-h wastewater incubation of pristine nanoparticles alone may be sufficient to prepare a more relevant model “weathered nanoparticle” sample that can be used for environmental studies seeking to inform future decision making. In future studies, further transformations on the weathered AgNPs through scenarios that mimic wastewater and biosolid treatment processes will be explored. 

## Figures and Tables

**Table 1:** ICP-MS result of Ag content in silver nanoparticle (AgNP) containing socks before any use or wastewater exposure.

Sample	mg/g of Sock
Commercial socks	0.0063 ± 0.0040
Laboratory-prepared socks	2.84 ± 0.47

**Table 2:** Mean concentration (±standard deviation), derived from triplicates, as measured through ICP-MS for Ag extracted from socks into the solution due to day-to-day use.

	Ag Concentration		
Day-To-Day Use	Sweat	Bleach	Detergent
Commercial socks (ng/L) (± 5 ng/L)	202	5	158
Laboratory-prepared socks (mg/L) (± 10 mg/L)	441	564	620

**Table 3:** Summary of the type of morphological changes and chemical associations of AgNPs when pristine or embedded into fabric, and applied to various use and exposure conditions.

Samples	Conditions	Changes		
		Agglomeration [23]	Aggregation [23]	Elemental Association
Laboratory-prepared Socks	Sweat	No	No	Cl, S
	Bleach	Yes	Yes	Cl
	Detergent	No	No	Cl, S
	Sweat + Wastewater	Yes	Yes	Cl, S
	Bleach + Wastewater	Yes	Yes	Cl, S
	Detergent + Wastewater	Yes	Yes	Cl, S
Pristine Nanoparticles	Sweat	No	Yes	Cl
	Bleach	Yes	Yes	Cl
	Detergent	No	Yes	Cl, S
	Sweat + Wastewater	Yes	Yes	Cl, S
	Bleach + Wastewater	Yes	Yes	Cl, S
	Detergent + Wastewater	Yes	Yes	Cl, S

## Supplementary Materials

The following are available online at https://www.mdpi.com/2079-4991/9/9/1258/s1, Figure S1: TEM image and EDS data of extracted samples from laboratory-prepared socks exposed to (a) sweat, (b) bleach, and (c) detergent. Circles indicate areas probed for EDS, Figure S2: TEM image and EDS data of pristine NPs weathered with (a) sweat, (b) bleach, and (c) detergent. Circles indicate areas probed for EDS, Figure S3: TEM image and EDS data of extracted samples from (a) sweat, (b) bleach, and (c) detergent solutions after treatment with wastewater. Circles indicate areas probed for EDS, Figure S4: TEM image and EDS data of pristine NPs treated with wastewater after weathering with (a) sweat, (b) bleach, and (c) detergent. Circles indicate areas probed for EDS, Figure S5: TEM image and EDS data of pristine AgNPs in (a) wastewater, (b) sulfur-rich solution, (c) nitrogen-rich solution, (d) wastewater + sulfur-rich solution, and € wastewater + nitrogen-rich solution. Circles indicate areas probed for EDS, Figure S6: TEM image and EDS data of pristine AgNPs in wastewater with pH altered to (a) pH 4.0, (b) pH 6.0, and (c) pH 8.0. Circles indicate areas probed for EDS, Table S1: The test results for raw wastewater composition. Sample 1 of raw wastewater was used for the pristine NPs. Sample 2 of raw wastewater was used for the solutions from day-to-day wear of laboratory-prepared socks and pristine NPs.
