# Supplementary material for: Morphological Transformation of Silver Nanoparticles from Commercial Products: Modeling from Product Incorporation, Weathering through Use Scenarios, and Leaching into Wastewater
Source: Nanomaterials (Basel). 2019 Sep 5;9(9):1258. doi: 10.3390/nano9091258 (PMC6781014; doi:10.3390/nano9091258)
Supplement: Supplementary file 1 [file nanomaterials-09-01258-s001.pdf]

Supporting Information

Table S1: The test results for raw wastewater composition. Sample 1 of raw wastewater was used for the pristine NPs. Sample 2 of raw waste water was used for the solutions from day-to-day wear of laboratory-prepared socks and pristine NPs.

Test	Units	Sample 1	Sample 2
Chemical Oxygen Demand, COD	mg/L	434	495
Total Nitrogen, N	mg/L N	47	51
Total Phosphorus, P	mg/L PO_4^{3-}	28	42
Total Sulfate, S	mg/L SO_4-	60	87
Ammonia, NH_3-N	mg/L NH_3-N	30.9	33
Nitrate, NO_3-N	mg/L NO_3^--N	0.9	1*
Turbidity	NTU	35	84
Absorbance @254nm	-	1.0489	1.5692
Total Solids	mg/L	1655.6	1072.5
Total Volatile Solids	mg/L	1189	392

*Test performed the following day

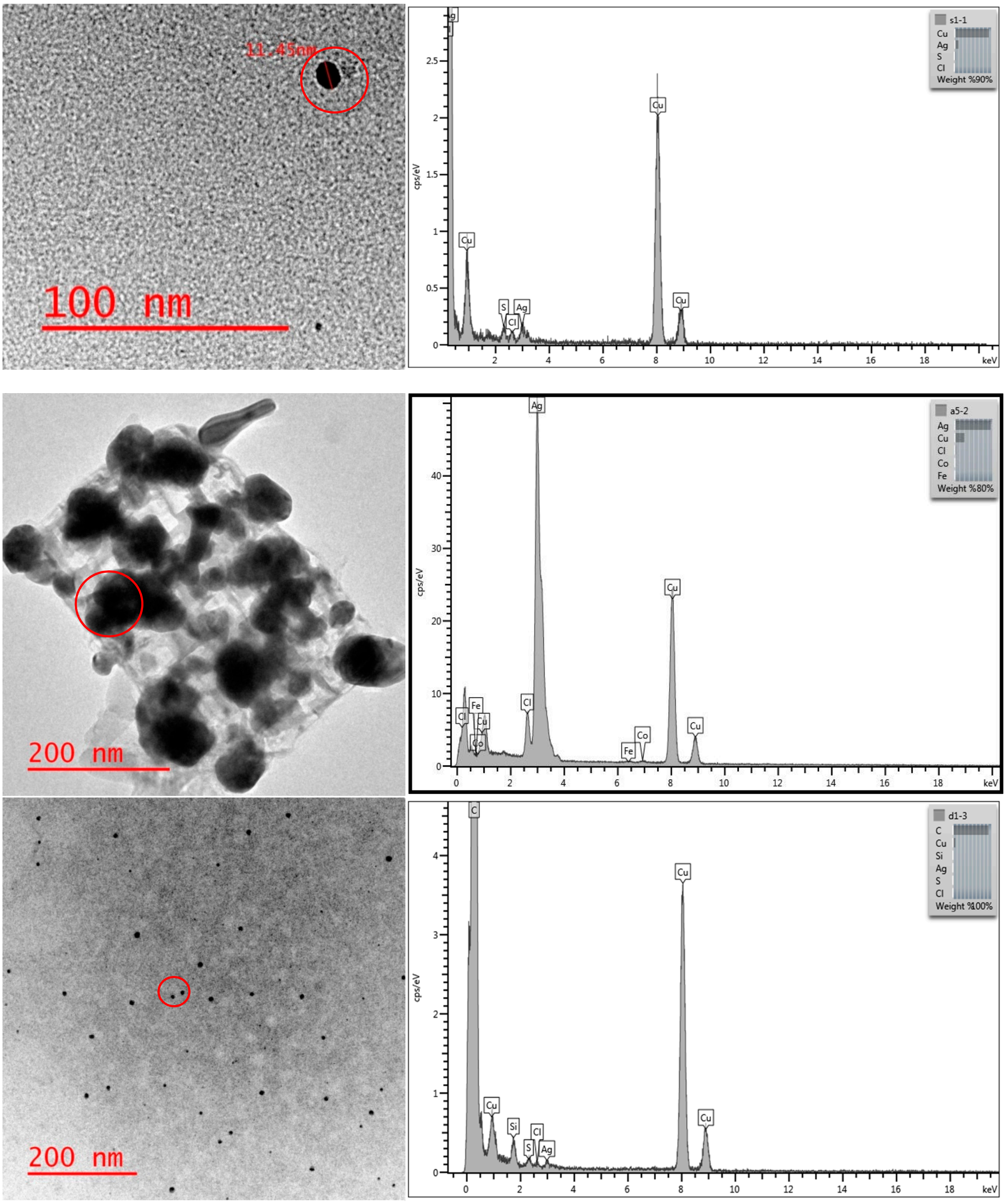

Figure S1: TEM image and EDS data of extracted samples from laboratory-prepared socks exposed to a) sweat b) bleach, c) detergent. Circles indicate areas probed for EDS.

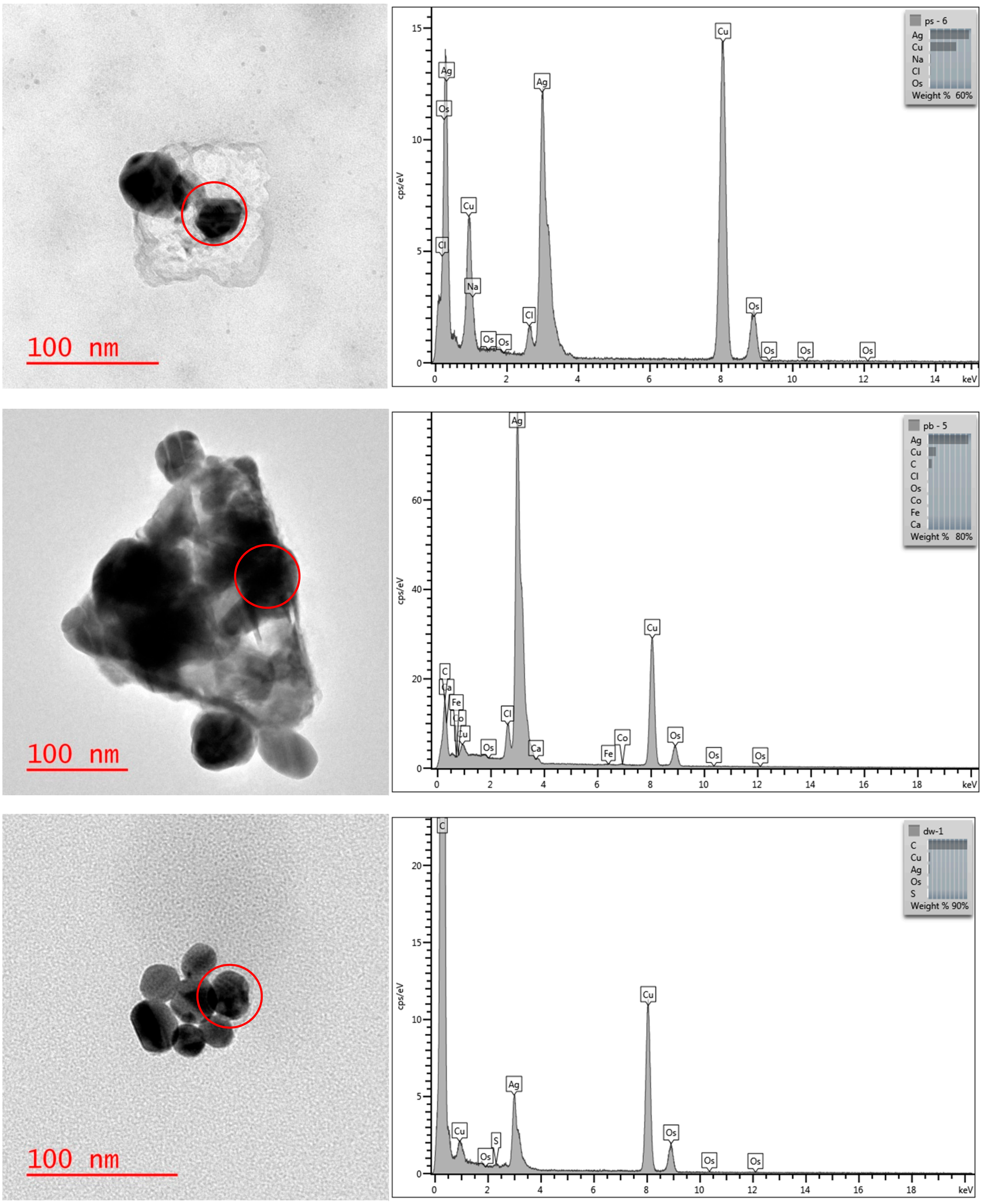

Figure S2: TEM image and EDS data of pristine NPs weathered with a) sweat b) bleach and c) detergent. Circles indicate areas probed for EDS.

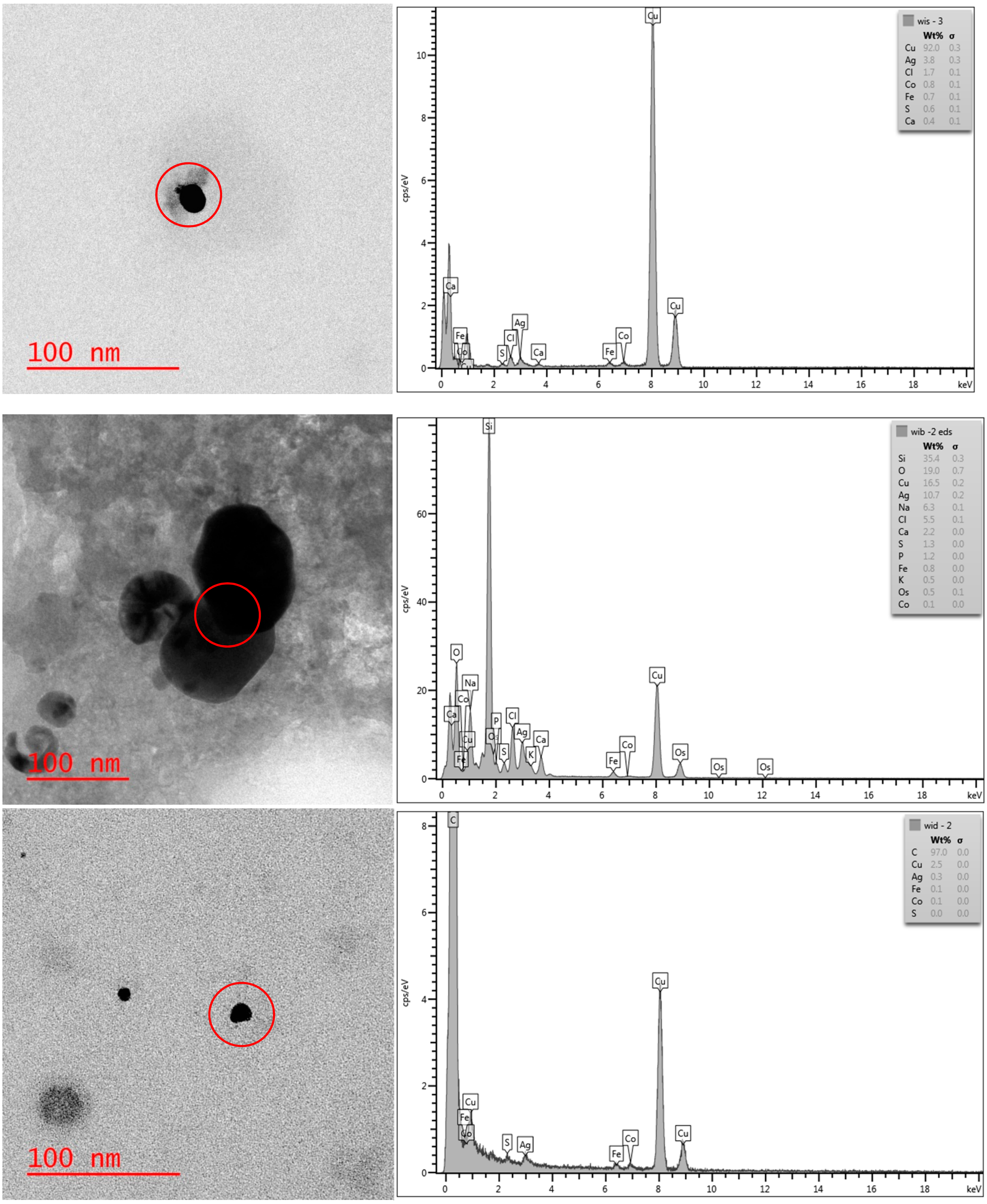

Figure S3: TEM image and EDS data of extracted samples from a) sweat b) bleach c) detergent solutions after treatment with wastewater. Circles indicate areas probed for EDS.

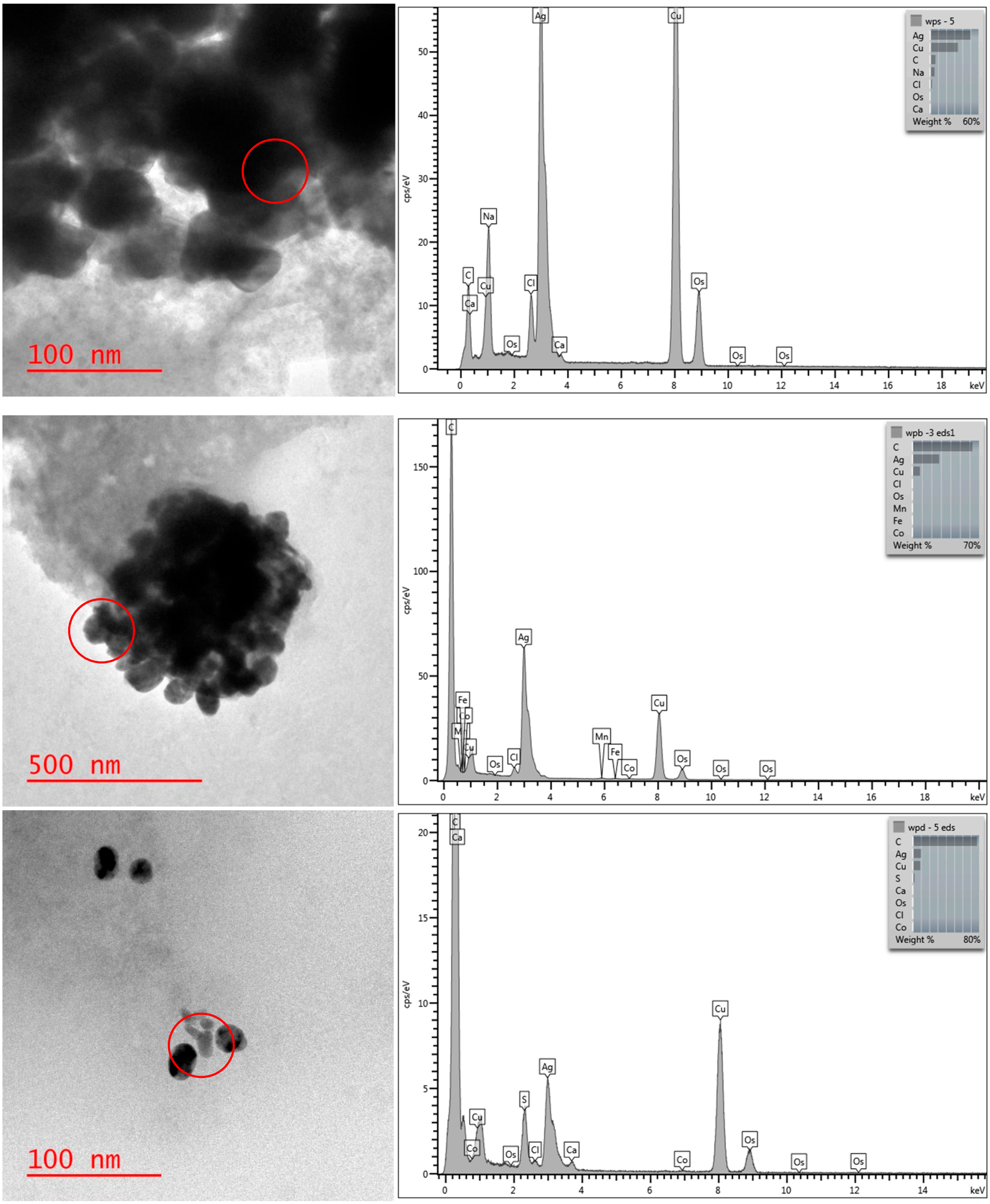

Figure S4: TEM image and EDS data of pristine NPs treated with wastewater after weathering with a) sweat b) bleach c) detergent. Circles indicate areas probed for EDS.

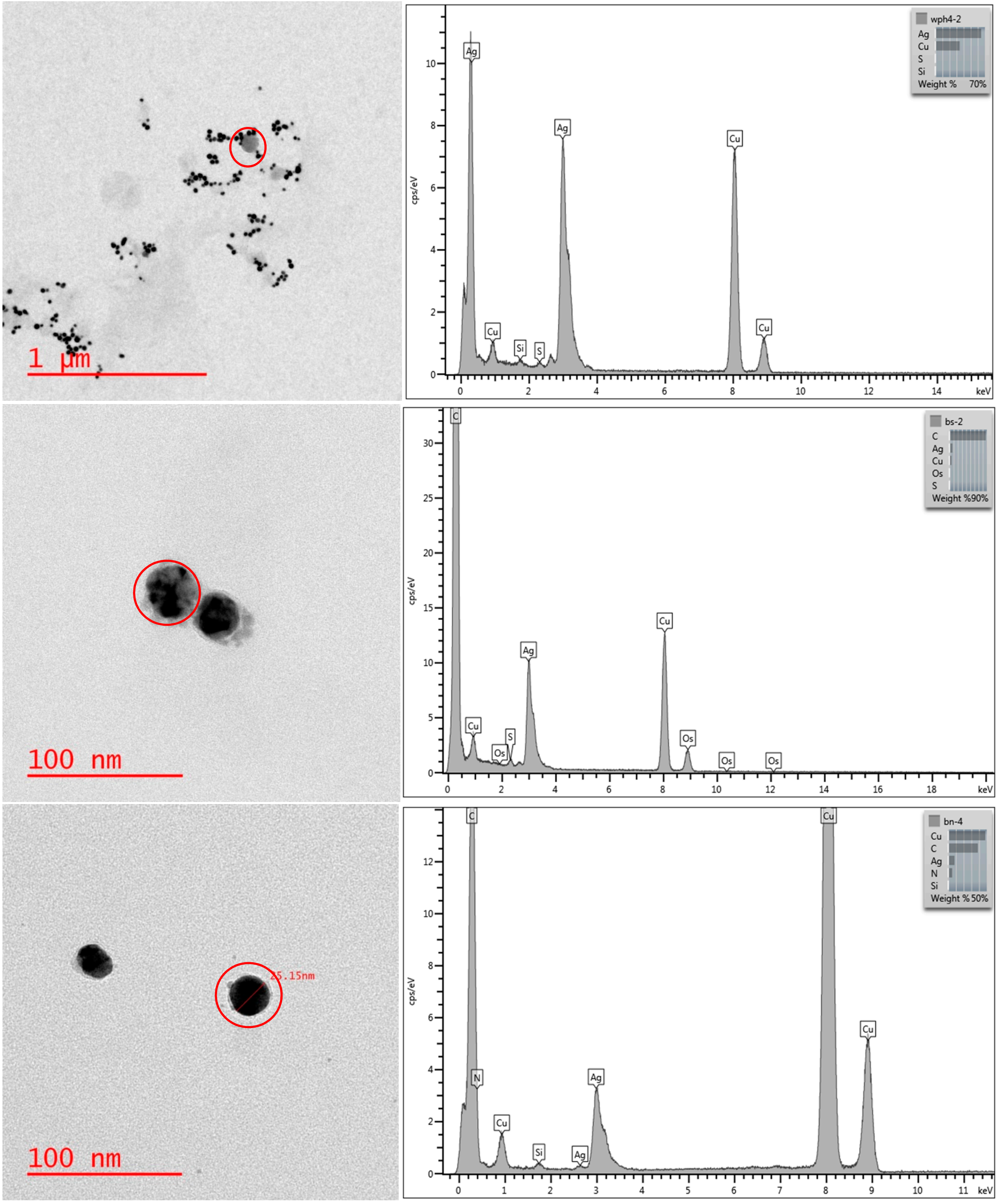
1 µm
wph4-2
Ag
Cu
S
Si
Weight % 70%
cps/eV
keV
100 nm
bs-2
C
Ag
Cu
Os
S
Weight %90%
cps/eV
keV
100 nm
25.15nm
bn-4
Cu
C
Ag
N
Si
Weight % 50%
cps/eV
keV

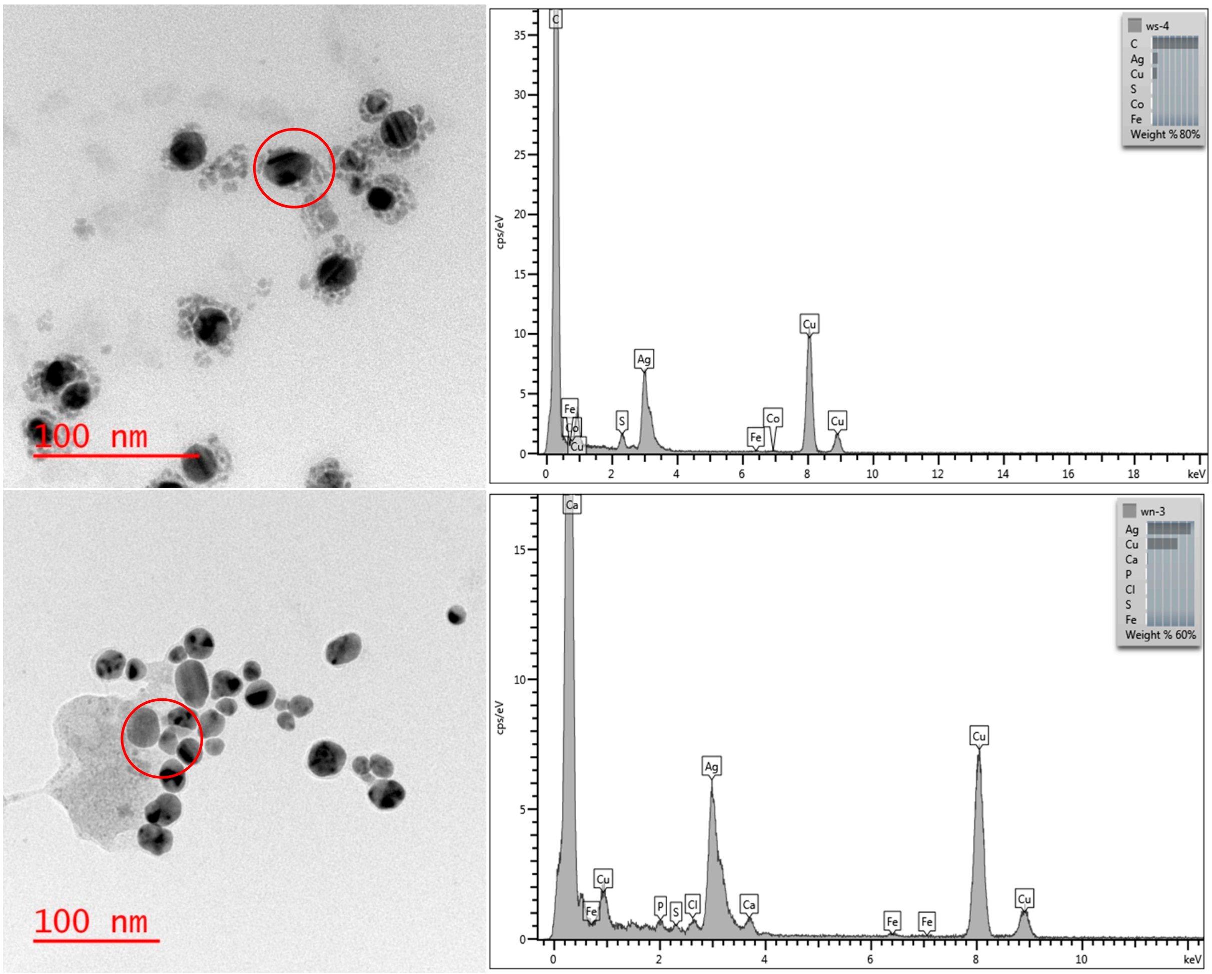

Figure S5: TEM image and EDS data of pristine AgNPs in a) wastewater b) sulfur-rich solution c) nitrogen-rich solution d) wastewater + sulfur-rich solution e) wastewater + nitrogen-rich solution. Circles indicate areas probed for EDS.

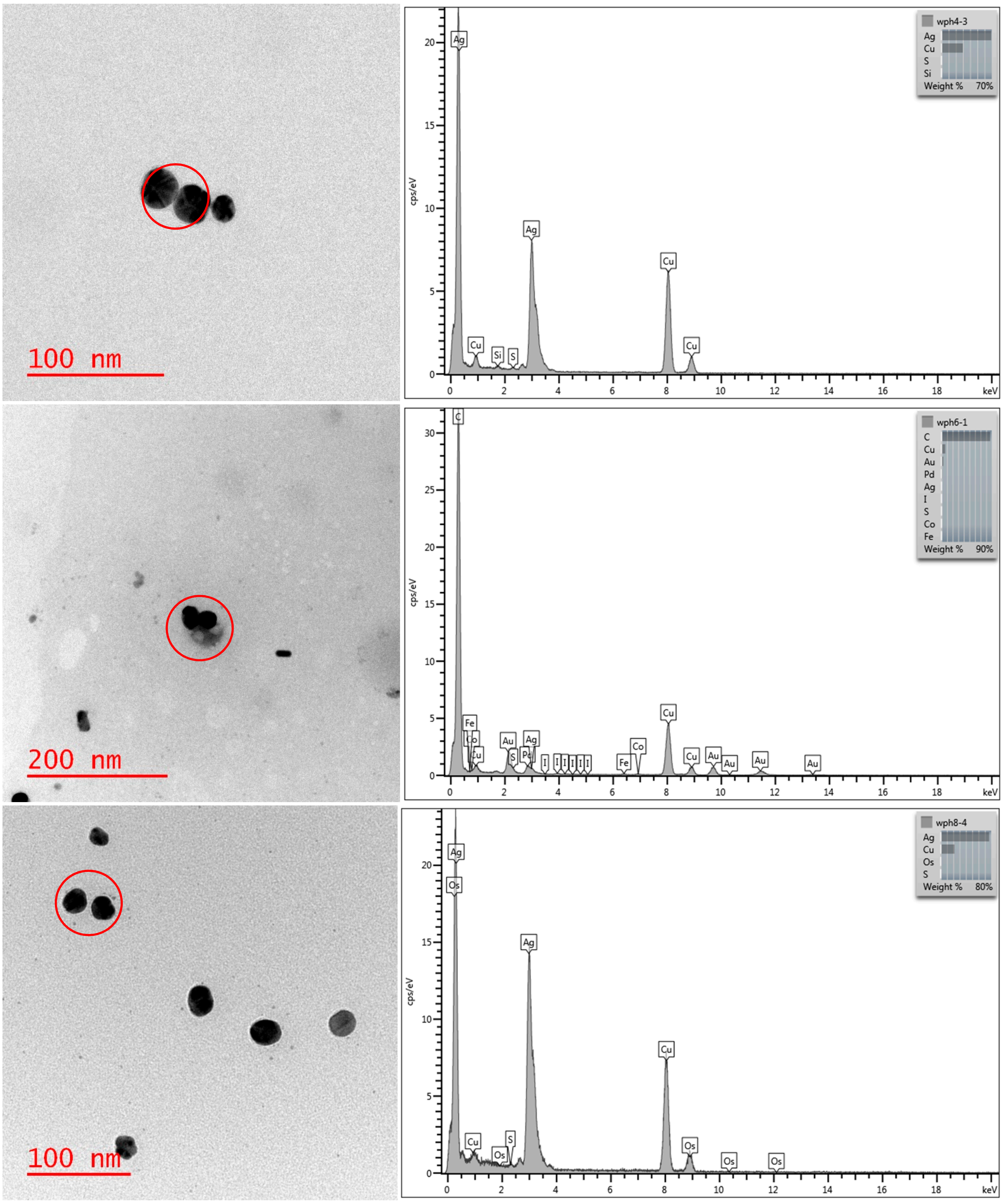

Figure S6: TEM image and EDS data of pristine AgNPs in wastewater with pH altered to a) pH 4.0 b) pH 6.0 c) pH 8.0. Circles indicate areas probed for EDS.
